# Associations Between Cognitive Functions and Subsequent Mood Disorder Prognosis in Low‐Risk, High‐Risk and Affected Monozygotic Twins: A Seven‐Year Follow‐Up Study

**DOI:** 10.1111/acps.70025

**Published:** 2025-08-20

**Authors:** Kamilla Miskowiak, Hanne Lie Kjærstad, Stella Lystlund, Anjali Sankar, Lars Kessing, Maj Vinberg

**Affiliations:** ^1^ Neurocognition and Emotion in Affective Disorders (NEAD) Centre, Psychiatric Centre Copenhagen Frederiksberg Hospital Frederiksberg Denmark; ^2^ Department of Clinical Medicine University of Copenhagen Copenhagen Denmark; ^3^ Copenhagen Affective Disorder Research Centre (CADIC), Psychiatric Centre Copenhagen Frederiksberg Hospital Frederiksberg Denmark; ^4^ The Neurobiology Research Unit Copenhagen University Hospital Copenhagen Denmark; ^5^ The Early Multimodular Prevention and Intervention Research Institution (EMPIRI), mental Health Centre North Zealand Copenhagen University Hospital, Mental Health Services in the Capital Region of Denmark Copenhagen Denmark

**Keywords:** at‐risk, cognition, longitudinal, mood disorders, onset, prognosis, progression

## Abstract

**Introduction:**

Aberrant cognition is common among individuals at familial risk for mood disorders (MD) and those already affected. However, long‐term prospective studies are needed to determine whether specific cognitive features predict illness onset and relapse; and whether cognitive impairments reflect neurodevelopmental traits or neuroprogressive decline.

**Methods:**

This seven‐year prospective study examined the relationship between cognition and illness progression in monozygotic twins with mood disorders, their healthy high‐risk monozygotic co‐twins, and low‐risk twins without a family history. Emotional and non‐emotional cognition was assessed at baseline (*n* = 204) and follow‐up (*n* = 124). Cox regression models tested whether baseline cognition predicted future illness onset in unaffected individuals (*n* = 89) or relapse in affected ones (*n* = 112). Longitudinal cognitive changes were analyzed using mixed models.

**Results:**

Greater attentional vigilance toward consciously processed happy faces at baseline was associated with a reduced risk of both illness onset (Exp(B) = 0.995, CI [0.990; 1.000], *p* = 0.03) and relapse (Exp(B) = 0.997, CI [0.995; 0.999], *p* = 0.003). Paradoxically, better verbal fluency at baseline was linked to an increased risk of illness onset (Exp(B) = 1.589, CI [1.204; 2.097], *p* < 0.001). Over time, onset was associated with increasing avoidance of subliminal fearful faces (group‐by‐time interaction, *p* < 0.001), whereas avoidance decreased in those who remained well. Verbal fluency declined in twins who developed a mood disorder (*p* = 0.02) but remained stable in those who stayed unaffected. No significant longitudinal cognitive differences emerged between affected twins with and without relapse.

**Conclusions:**

Positive attentional biases may protect against illness onset and relapse, while greater baseline verbal fluency may unexpectedly signal vulnerability. Verbal fluency decline after illness onset likely reflects scar effects. The findings underscore the importance of early identification of cognitive‐emotional vulnerabilities and suggest targets for preventive interventions.

**Summary**

- Significant outcomes
  ○ Increased vigilance toward consciously processed happy faces is associated with a lower risk of mood disorder onset and relapse.
  ○ Greater avoidance of non‐consciously processed fearful faces emerges following mood disorder onset.
  ○ Higher baseline verbal fluency is associated with subsequent onset of mood disorders, after which verbal fluency declines.
- Limitations
  ○ Moderate sample size for follow‐up cognitive assessments.
  ○ No correction for multiple comparisons, reflecting the exploratory nature of the study.
  ○ Non‐emotional cognition was assessed with only a brief cognitive screening tool.

## 1 Introduction

Major depression disorder (MDD) and bipolar disorder (BD), collectively referred to as ‘mood disorders’, are disabling mental health conditions characterised by an episodic and highly recurrent pattern of illness, including episodes of depression and/or (hypo)mania [1]. Individuals diagnosed with mood disorders exhibit markedly elevated morbidity rates [2, 3] and an increased risk of suicide [4], leading to a life expectancy that is 8–12 years shorter than that of the general population [5]. As a result, mood disorders rank among the top contributors to the global disease burden [6]. Despite treatment efforts, many individuals with mood disorders do not achieve or maintain symptomatic remission [7, 8, 9]. Indeed, the initial mood episode often prompts a progressive illness course characterised by high relapse rates and increasingly shorter intervals between mood episodes ([10]) as well as persistent reductions in the psychosocial and occupational functioning [11, 12, 13]. Identifying objective illness biomarkers is crucial to enhance prophylactic and early intervention strategies, enabling the prediction of illness onset in at‐risk individuals and prognosis in affected individuals [14].

Endophenotypes are measurable, hereditary, and state‐independent indicators of illness, evident in those experiencing symptomatic remission and unaffected relatives [15]. Aberrant emotional and non‐emotional cognition has been suggested as potential endophenotypes of mood disorders, with deficits being present throughout symptomatically stable periods of remission in affected individuals [16, 17, 18, 19] and in their unaffected first‐degree relatives [20, 21] compared to the general population. Several studies found aberrant emotional cognition, including emotional face processing, in healthy individuals at familial risk of mood disorders and affected individuals in remission [22, 23]. Prospective studies on cognitive predictors of illness onset and prognosis in mood disorders are limited. However, several studies in BD associate general cognitive impairment, poorer verbal memory, executive dysfunction, and positive bias with subsequent (hypo)manic—but not depressive—relapse [24]. In addition, poorer verbal memory is associated with future hospitalisation risk in individuals with MDD and BD [25]. In first‐degree relatives, deficits in attention, verbal memory, executive function, and positive bias were associated with future illness onset [24]. Together, this provides emerging evidence for aberrancies in emotional and non‐emotional cognition in at‐risk and affected individuals that may impact their long‐term prognosis.

Cross‐sectional studies suggest a relationship between cognition and clinical markers of illness progression, that is, the number of prior mood episodes, indicative of neuroprogression [17, 18]. However, longitudinal studies found that cognitive deficits in patients with recent onset [26, 27] were stable across both shorter [28, 29] and longer [28, 30] periods. Emerging evidence suggests that cognitive trajectories differ among individuals with mood disorders, with manic episodes being associated with greater cognitive decline over time [31, 32]. There are two key hypotheses regarding the mechanisms underlying cognitive impairment in mood disorders that emphasise either *neuroprogressive* and *neurodevelopmental* origins, respectively. The neuroprogression hypothesis suggests that mood episodes lead to structural and functional brain changes that result in a progressive decline of cognitive functioning from early to more chronic illness stages [33, 34, 35]. In contrast, the neurodevelopmental hypothesis posits cognitive deficits as trait‐related manifestations of abnormalities in early developmental neurobiological processes [33].

Key methodological challenges of extant longitudinal studies include (i) short follow‐up times, with around 67% of studies having a test–retest period of 3–6 months [28]; (ii) small sample sizes; (iii) no inclusion of individuals at familial risk; and (iv) the absence of emotional cognition measures. To clarify the neuroprogressive versus neurodevelopmental origins of cognitive changes in mood disorders, long‐term prospective studies in affected individuals and high‐risk populations are needed. Such studies will clarify whether cognitive impairment in mood disorders reflects progressive disease‐related decline or stable, genetically predisposed neurodevelopmental deficits and help to determine whether cognition can predict illness onset and relapse.

## 2 Aims of the Study

This study aimed to investigate cognitive functioning in a seven‐year longitudinal cohort study of affected or unaffected at‐risk twins, and healthy low‐risk monozygotic twins, focusing on associations between cognitive performance and mood episodes. Our study had two objectives: First, to examine whether baseline cognitive performance is associated with future relapse in affected twins and illness onset in high‐ and low‐risk twins. Second, to investigate whether mood episodes were linked to cognitive changes over time by comparing (i) affected twins who relapsed versus those who remained in remission and (ii) unaffected twins who later developed illness versus those who remained unaffected.

## 3 Materials and Methods

### 3.1 Study Design and Participants

This longitudinal study examined cognitive trajectories over 7 years in monozygotic twins from the Neuromapping of Endophenotypes for Affective Disorders (NEAD) cohort [36]. Participants were assessed twice: baseline assessments occurred from December 2014 to January 2017, and follow‐up assessments from July 2022 to April 2024. The study was approved by the Danish National Board of Health, the Data Protection Agency (Approval No. 2014‐331‐0751), and the Regional Ethics Committee (Approval Nos. H‐3‐2014‐003, H‐22022286). All participants provided written informed consent, and assessors were blinded to participants' diagnostic status during cognitive testing.

A total of 476 eligible monozygotic twins were identified through linkage between the Danish Twin Registry, the Psychiatric Central Research Register, and the Civil Registration System. Of 408 invited, 209 participated after excluding individuals for low birth weight (< 1.3 kg), severe medical illness, brain injury, active substance abuse, ongoing mood episodes (HDRS‐17 or YMRS > 14), pregnancy, or schizophrenia spectrum diagnoses. Psychiatric diagnoses were confirmed using the Schedules for Clinical Assessment in Neuropsychiatry (SCAN) [37]. The final baseline sample included 204 participants (115 affected, 49 high‐risk and 40 low‐risk), with 89 complete twin pairs. At follow‐up, information regarding episode relapse and/or onset over the follow‐up time was available for 201 monozygotic twins (112 affected, 49 high‐risk and 40 low‐risk), whereas cognition data was obtained for 124 monozygotic twins (67 affected, 33 high‐risk and 24 low‐risk) [38].

### 3.2 Procedures

Baseline and follow‐up visits included biological sampling, mood and functioning assessments, and 2 h of cognitive testing. Follow‐up diagnostic interviews were conducted using the Mini‐International Neuropsychiatric Interview (M.I.N.I.) [39]. Functioning was measured with the Functioning Assessment Short Test (FAST) (Rosa et al. 2007). Subsyndromal mood symptoms were assessed using the HDRS‐17 [40] and the YMRS [41]. Mood episodes occurring between baseline and follow‐up were verified via M.I.N.I. and SCAN to confirm episodes for respondents or with registry data for non‐respondents, defined as hospital admissions for mood disorders recorded in the Psychiatric Central Register (data available until March 2022).

### 3.3 Cognitive Assessments

Emotional cognition was assessed with the Oxford Emotional Test Battery [42], including the Faces Dot‐Probe Task, Facial Expression Recognition, Emotional Categorization, and Recall (based on Anderson, 1968), and the Social Scenarios Task [43]. For details on these tests, see the Supporting Information.

Non‐emotional cognitive functioning was assessed using the Screening for Cognitive Impairment in Psychiatry (SCIP) [44], Trail Making Test [45], and the Danish Adult Reading Test (DART) [45].

### 3.4 Psychological Questionnaires

Participants completed the Childhood Trauma Questionnaire (CTQ; Bernstein et al. [46]), Eysenck Personality Questionnaire (EPQ; Eysenck and Eysenck [47]), the Coping Inventory of Stressful Situations (CISS) [48], the Stressful Life Events questionnaire (SLE; Kendler et al. [49]), and the State–Trait Anxiety Inventory (STAI) [50]. The CTQ and Social Scenarios Task were administered only at baseline.

### 3.5 Statistical Analyses

For the Faces Dot‐Probe task, attentional bias scores were calculated by subtracting response times in congruent from incongruent trials, with positive values indicating vigilance and negative values indicating avoidance. Facial expression recognition response times were *z*‐transformed per time point due to different testing devices and truncated at ±4 SD from the low‐risk twin mean to minimize outlier effects. Discrimination accuracy (*d*′) was calculated, and accuracy and response times were averaged for positive (happiness and surprise) and negative (anger, fear, disgust, and sadness) expressions. For the Social Scenarios Task, emotion regulation success was calculated as the difference between ratings in passive view and down‐regulation conditions. SCIP scores were standardized using normative data from a healthy sample (*n* = 273; [51]), adjusting for sex, age, and education.

To assess whether baseline cognitive performance predicted mood episode onset or relapse, Cox regression was used with baseline hazard stratified by group (affected, high‐risk, low‐risk). Analyses adjusted for sex, age, mood symptoms, and prior episodes (for relapse models) and accounted for clustering within twin pairs. Three types of analyses were conducted, associating baseline cognition with (i) the risk of onset in those unaffected at baseline, (ii) the risk of relapse among those affected with mood disorder at baseline, and (iii) the overall risk of an event. Due to a limited number of events in the low‐risk group, the low‐risk and high‐risk groups were collapsed into one group, categorized as ‘unaffected’ at baseline, while still stratifying by group to account for the higher probability of an event in the high‐risk group.

To assess the secondary aim, whether mood episodes were associated with changes in cognitive performance over time, we investigated differential changes in cognition in (i) affected twins who relapsed during the follow‐up period compared to affected twins who remained in remission; and (ii) unaffected twins (i.e., both low‐risk and high‐risk) who later developed illness vs. those who remained unaffected. Changes in cognition over time were examined using mixed‐model analyses with group and time as fixed factors and familial linkage as a random factor to account for co‐variance within twin pairs. When significant group‐by‐time interactions were identified, additional pairwise mixed models were conducted. Continuous demographic, clinical, and cognitive variables were compared using mixed models with group as a fixed factor and twin pairs as a random factor. Dichotomous variables were analyzed using the chi‐square. Due to the study's exploratory nature, analyses were not adjusted or corrected for multiple comparisons.

## 4 Results

### 4.1 Participants and Demographics

At baseline, 204 monozygotic twins were included: 115 affected, 49 high‐risk, and 40 low‐risk [52]. At follow‐up (7.0 ± 0.8 years later), 124 monozygotic twins were re‐assessed (67 affected, 33 high‐risk and 24 low‐risk) (Table S1). For analyses comparing affected, high‐risk, and low‐risk twins, see Supporting Information. For participants who did not participate in the follow‐up assessments (*n* = 71), information on mood episodes was obtained from national registers (see Sperling et al. [38], for details).

During the follow‐up period, 19 (21%) participants who were unaffected at baseline developed a mood disorder (high‐risk *n* = 16; low‐risk *n* = 3); while the remaining 70 (79%) (high‐risk *n* = 33; low‐risk *n* = 37) remained unaffected.

**FIGURE 1:**
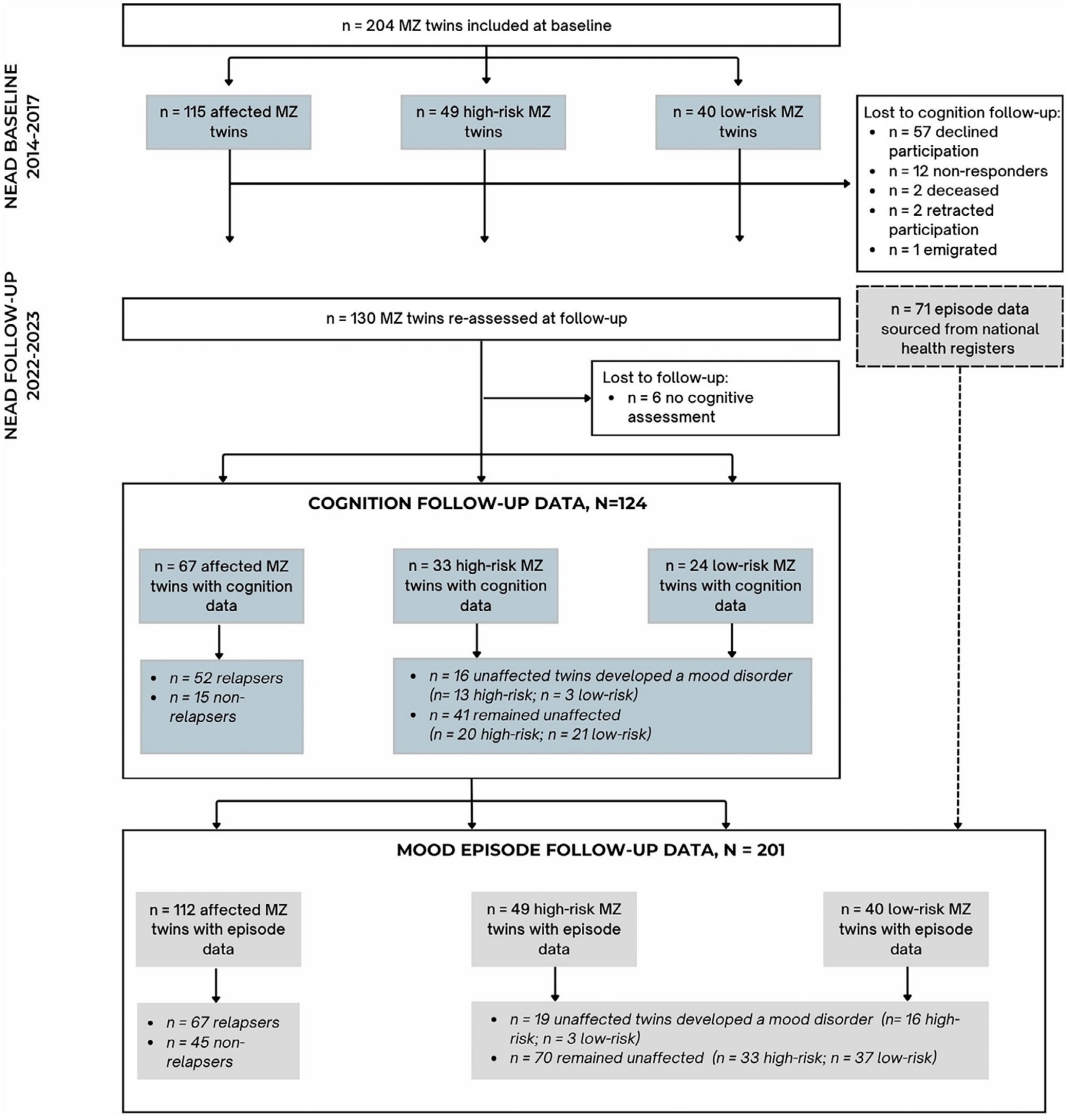
Flowchart depicting participant flow from baseline to follow‐up cognitive assessments.

Cognitive follow‐up data were available for 16 unaffected relatives who developed a mood disorder and 41 who remained unaffected. Additionally, among the affected twins, 67 (60%) experienced a relapse during the follow‐up, while 45 (40%) remained in remission. Follow‐up cognitive data were available for 52 of the affected twins who relapsed and 15 of those who remained in remission (see Figure 1 for flowchart). Follow‐up duration did not differ significantly between unaffected twins with and without subsequent onset (*p* = 0.81) or affected twins with and without a subsequent relapse (*p* = 0.15). For comparative analyses of drop‐outs and follow‐up participants, see the Supporting Information.

### 4.2 Baseline Characteristics

Unaffected twins with subsequent illness onset and those who remained healthy showed no significant differences in demographics, IQ, symptoms, functioning, or childhood trauma (*p* levels ≥ 0.15) except for higher task‐oriented coping in the onset group (*p* = 0.04) (see Table 1). There was no significant difference between unaffected twins with and without subsequent onset in baseline emotional (*ps* ≥ 0.08) or non‐emotional cognition (*ps* ≥ 0.12) (see Table 2).

**TABLE 1:** Demographics and clinical variables in unaffected monozygotic twins with and without illness onset at baseline and follow‐up.

	Baseline				Follow‐up				Group‐by‐time interaction
	Onset (*n* = 19)	Remained unaffected (*n* = 70)	*F*/*χ*	*p*	Onset (*n* = 16)	Remained unaffected (*n* = 41)	*F*/*χ*	*p*	*p*
Demographics									
Sex, *n* (% female)	14 (74)	51 (73)	0.01	0.94					
Age, years	37.1 (9.3)	37.0 (9.4)	0.00	0.98	43.9 (9.0)	45.7 (8.5)	0.00	1.00	
Education, years	15.9 (3.2)	15.4 (2.8)	0.28	0.60	16.2 (3.2)	16.0 (2.5)	0.12	0.73	
Verbal full‐scale IQ	114.7 (5.8)	112.8 (6.4)	1.24	0.27					
Functioning									
FAST total	3.6 (3.5)	3.3 (5.0)	0.44	0.51	12.6 (8.1)	12.9 (9.4)	0.004	0.95	0.74
FAST autonomy	0.4 (1.2)	0.3 (0.7)	0.06	0.81	2.0 (2.0)	1.5 (1.7)	1.29	0.26	0.54
FAST occupation	0.3 (0.9)	0.3 (1.8)	0.02	0.90	0.9 (1.2)	2.7 (4.5)	2.54	0.12	0.11
FAST cognition	1.5 (1.4)	1.2 (1.8)	0.72	0.40	3.3 (2.5)	2.5 (2.5)	1.14	0.29	0.84
FAST financial	0.1 (0.5)	0.2 (0.5)	0.35	0.56	0.3 (0.6)	0.7 (1.0)	1.57	0.22	0.33
FAST interpersonal	0.9 (1.3)	1.0 (1.7)	0.51	0.48	4.3 (2.7)	3.5 (2.6)	1.34	0.25	0.22
FAST leisure	0.5 (0.9)	0.4 (0.8)	0.43	0.51	1.9 (1.3)	2.0 (1.5)	0.01	0.93	0.92
Clinical variables									
High risk, *n* (%)	16 (84)	33 (47)							
MDD, *n* (%)	10 (53)	28 (40)							
BD, *n* (%)	6 (32)	5 (7)							
HDRS	2.8 (2.2)	2.2 (2.4)	1.49	0.23	3.2 (3.0)	2.2 (3.0)	1.20	0.28	0.37
YMRS	1.6 (1.1)	1.3 (1.5)	0.22	0.64	1.4 (2.3)	0.4 (1.4)	3.80	0.07	0.22
STAI‐state	29.3 (7.7)	27.6 (6.6)	0.76	0.39	30.7 (5.4)	28.7 (8.7)	0.54	0.47	0.89
STAI‐trait	35.7 (4.8)	33.7 (5.8)	1.86	0.18	36.2 (6.7)	34.7 (7.3)	0.53	0.47	0.43
Neuroticism	7.6 (4.3)	6.8 (4.8)	0.17	0.68					
Childhood Trauma Questionnaire (CTQ)									
Total	35.9 (11.1)	32.0 (10.8)	0.00	0.96					
Physical abuse	5.5 (1.4)	5.6 (1.8)	2.26	0.15					
Emotional abuse	8.9 (4.4)	7.4 (3.8)	0.41	0.52					
Sexual abuse	3.8 (1.0)	4.0 (1.3)	0.26	0.61					
Emotional neglect	11.2 (5.1)	8.6 (4.3)	0.27	0.61					
Physical neglect	6.5 (2.6)	6.4 (2.6)	0.01	0.91					
Stressful Life Events (SLE) and Coping in Stressful Situations (CISS)									
SLE before	2.2 (2.0)	2.1 (1.6)	0.37	0.55	2.1 (1.4)	2.1 (1.6)	0.12	0.73	0.42
Task‐oriented	61.4 (8.9)	55.0 (11.5)	4.54	0.**04**	59.7 (9.5)	57.1 (10.8)	0.51	0.48	0.55
Emotion‐oriented	36.6 (9.5)	36.3 (12.6)	0.13	0.72	34.7 (11.0)	35.1 (11.1)	0.07	0.80	0.88
Avoidance	39.1 (6.8)	39.0 (8.7)	0.69	0.41	37.3 (8.5)	37.7 (8.7)	0.01	0.91	0.93
Distraction	14.2 (4.6)	14.9 (4.7)	0.02	0.89	15.1 (4.6)	14.8 (4.9)	0.08	0.78	0.57
Social diversion	17.2 (4.5)	16.4 (4.4)	0.77	0.38	15.5 (5.3)	16.5 (4.8)	0.47	0.50	0.30

*Note:* the sample of *unaffected* monozygotic twins includes twins who were at low‐ and high‐risk of affective disorders at baseline. Of these, *n* = 19 (3 low‐risk and 16 high‐risk) developed illness onset during the follow‐up, and *n* = 70 (37 low‐risk and 33 high‐risk) remained unaffected. Cognition data at follow‐up was available for *n* = 16 of the unaffected monozygotic twins who developed illness onset, and *n* = 40 of those who remained unaffected. Scores are indicated in mean (standard deviation). Bold values indicate significant values. FAST = Functional Assessment Short Test; HDRS = Hamilton Depression Rating Scale; YMRS = Young Mania Rating Scale.

**TABLE 2:** Cognition in unaffected monozygotic twins with and without illness onset.

	Baseline				Follow‐up				Group‐by‐time interaction
	Onset (*n* = 19)	Remained unaffected (*n* = 70)	*F*/*χ*	*p*	Onset (*n* = 16)	Remained unaffected (*n* = 41)	*F*/*χ*	*p*	*p*
Emotional cognition									
DotProbe Faces Task									
Masked fear	−4.1 (98.9)	−40.1 (75.9)	2.63	0.11	−66.1 (55.4)	−9.0 (54.0)	10.48	0.**002**	**< 0.001**
Masked happy	−3.1 (121.8)	30.2 (154.1)	2.78	0.10	4.4 (88.7)	5.0 (55.1)	0.003	0.95	0.24
Unmasked fear	−28.9 (124.3)	−38.8 (83.3)	0.15	0.70	−30.7 (98.2)	−17.6 (50.4)	0.41	0.53	0.51
Unmasked happy	−15.1 (119.1)	33.2 (92.6)	3.08	0.08	42.4 (106.6)	38.2 (105.0)	0.01	0.91	0.20
Facial Expression Recognition Test (FERT)									
Discrimination accuracy (d')									
Positive emotions	0.5 (0.2)	0.5 (0.1)	0.17	0.69	0.5 (0.0)	0.5 (0.1)	0.09	0.77	0.53
Negative emotions	0.4 (0.2)	0.4 (0.1)	0.54	0.47	0.4 (0.1)	0.4 (0.1)	1.03	0.32	0.79
Response time, z									
Positive emotions	0.2 (0.8)	−0.2 (1.1)	1.62	0.21	0.2 (0.5)	−0.2 (1.1)	2.02	0.16	0.53
Negative emotions	0.2 (0.8)	−0.1 (1.1)	0.73	0.40	0.1 (0.7)	−0.0.2 (1.1)	0.77	0.25	0.63
Emotional Categorization and Recall Task									
Categorization accuracy (1 = 100%)									
Positive words	1.0 (0.0)	0.9 (0.1)	3.22	0.08	0.9 (0.0)	1.0 (0.1)	0.07	0.80	0.27
Negative words	0.9 (0.0)	0.9 (0.1)	0.02	0.90	0.9 (0.0)	0.9 (0.1)	0.09	0.76	0.54
Mean response time categorization, *z*									
Positive words	0.1 (0.9)	−0.1 (1.0)	0.12	0.73	0.1 (1.0)	0.2 (1.0)	0.01	0.91	0.37
Negative words	0.1 (1.0)	−0.1 (1.1)	0.21	0.65	0.0 (0.7)	0.2 (1.1)	0.29	0.59	0.07
Number of words recalled									
Positive words	3.9 (2.3)	3.9 (2.6)	0.01	0.94	3.9 (2.6)	3.3 (1.9)	0.61	0.44	0.45
Negative words	3.8 (2.7)	3.4 (2.2)	0.92	0.34	3.0 (2.0)	2.6 (2.1)	0.29	0.59	0.99
Social scenarios									
Emotion reactivity									
Positive emotions	77.9 (8.5)	78.0 (13.1)	0.13	0.72					
Negative emotions	70.1 (16.6)	69.5 (18.2)	0.01	0.94					
Emotion downregulation									
Positive emotions	19.8 (14.6)	16.4 (15.1)	0.76	0.39					
Negative emotions	26.1 (20.9)	22.9 (22.0)	0.42	0.52					
Non‐emotional cognition									
TMT‐A, s	29.0 (10.1)	29.0 (10.0)	0.12	0.73	31.4 (9.6)	29.3 (8.0)	0.95	0.34	0.58
TMT‐B, s	69.3 (28.0)	77.4 (31.0)	0.92	0.34	80.3 (33.0)	71.1 (20.3)	1.14	0.29	0.07
SCIP total^a^	−0.1 (1.4)	−0.4 (1.49)	0.11	0.74	−0.3 (1.3)	−0.1 (1.5)	0.27	0.61	0.66
VLT‐I^a^	0.1 (1.0)	−0.1 (1.4)	0.001	0.98	0.2 (1.0)	0.0 (1.7)	0.01	0.94	0.52
WMT^a^	−0.4 (1.1)	−0.4 (1.5)	0.05	0.83	−0.4 (1.2)	−0.2 (1.6)	0.21	0.65	0.83
VFT^a^	0.1 (1.1)	−0.3 (1.2)	2.11	0.15	−0.4 (1.1)	−0.2 (1.1)	0.78	0.38	0.**008**
VLT‐D^a^	0.2 (0.8)	−0.4 (1.3)	2.42	0.12	0.1 (1.0)	0.0 (1.3)	0.14	0.71	0.67
PST^a^	−0.5 (1.9)	0.1 (1.0)	1.67	0.20	−0.3 (1.0)	0.0 (1.0)	1.20	0.28	0.25

*Note:* Scores are indicated in mean (standard deviation) if otherwise is not specified. Bold values indicate significant values.

^a^ Number of SD from expected scores based on sex, age, and years of education.

Among affected twins, there was a trend toward more females in the relapse group (*p* = 0.051; Table 3), but no significant differences in clinical or functional characteristics, except for higher lifetime stress exposure in relapsers (*p* = 0.02). No significant difference was found between affected twins with and without subsequent relapse in baseline emotional (*ps* ≥ 0.12) or non‐emotional (*ps* ≥ 0.12) cognition (see Table 4).

**TABLE 3:** Demographics and clinical variables in affected monozygotic twins with and without illness relapse at baseline and follow‐up.

	Baseline				Follow‐up				Group‐by‐time interaction G
	Affected relapsers (*n* = 67)	Affected non‐relapser (*n* = 45)	*F*/*χ*	*p*	Affected relapsers (*n* = 52)	Affected non‐relapsers (*n* = 15)	*F*/*χ*	*p*	*p*
Demographics									
Sex, *n* (% female)	51 (77)	27 (60)	3.8	0.051					
Age, years	37.9 (8.5)	34.0 (8.6)	1.3	0.27	45.8 (7.8)	43.3 (9.9)	0.00	1.00	
Education, years	14.6 (3.5)	14.4 (3.0)	0.002	0.97	14.8 (3.6)	14.3 (2.8)	0.07	0.80	
Verbal full‐scale IQ	114.0 (6.4)	112.4 (6.3)	0.92	0.34					
Functioning									
FAST total	14.0 (11.0)	12.8 (12.5)	0.26	0.61	23.2 (14.0)	21.8 (13.9)	0.08	0.77	0.68
FAST autonomy	1.0 (1.6)	0.8 (1.7)	0.25	0.62	2.7 (2.5)	1.4 (2.6)	2.96	0.09	0.08
FAST occupation	6.0 (6.7)	4.8 (6.2)	0.81	0.37	7.7 (7.0)	7.2 (6.8)	0.03	0.87	0.98
FAST cognition	3.2 (2.5)	2.7 (2.7)	1.13	0.29	4.7 (3.4)	4.9 (3.2)	0.07	0.80	0.77
FAST financial	0.5 (1.0)	0.3 (1.1)	0.74	0.39	0.8 (1.4)	0.5 (0.7)	0.58	0.45	0.92
FAST interpersonal	2.4 (2.6)	3.2 (3.7)	1.79	0.18	5.2 (3.4)	5.5 (3.9)	0.04	0.84	0.97
FAST leisure	0.9 (1.4)	1.0 (1.4)	0.28	0.60	2.0 (1.8)	2.3 (1.7)	0.87	0.36	0.98
Clinical variables									
MDD, *n* (%)	47 (71)	34 (76)							
BD, *n* (%)	19 (29)	11 (24)							
BD type II, *n* (%)	4 (21)	5 (46)							
Onset age	23.8 (7.9)	23.0 (7.3)	0.03	0.86					
Total episodes	5.7 (10.0)	3.8 (9.0)	1.83	0.18					
Hospitalizations	2.9 (12.5)	1.5 (6.0)	0.19	0.67					
Suicide attempts	1.2 (6.3)	0.5 (1.7)	0.44	0.51					
HDRS	4.9 (3.5)	4.7 (3.7)	0.13	0.72	4.9 (3.7)	2.5 (2.2)	5.74	0.02	0.03
YMRS	2.1 (2.4)	1.4 (1.5)	2.86	0.09	1.3 (2.0)	0.8 (1.6)	0.60	0.44	0.49
STAI‐state	32.0 (7.7)	31.5 (7.4)	0.13	0.72	35.2 (9.4)	34.3 (8.9)	3.39	0.78	0.49
STAI‐trait	41.2 (8.4)	41.5 (9.0)	0.01	0.93	41.7 (8.2)	39.3 (9.3)	0.47	0.50	0.20
Neuroticism	12.1 (5.4)	11.6 (5.6)	0.20	0.65					
Medication, *n* (%)									
AD	27 (41)	16 (36)	0.32	0.57	14 (27)	6 (40)	0.95	0.33	
AP	12 (18)	6 (13)	0.46	0.50	3 (6)	2 (13)	0.97	0.33	
AE	13 (19)	4 (9)	2.41	0.12	6 (12)	1 (7)	0.30	0.59	
LI	9 (14)	3 (7)	1.35	0.25	4 (6)	0 (0)	1.23	0.27	
Childhood Trauma Questionnaire (CTQ)									
Total	39.6 (12.3)	35.4 (10.0)	1.59	0.21					
Physical abuse	5.9 (1.9)	5.7 (1.6)	0.17	0.68					
Emotional abuse	9.9 (4.5)	8.4 (3.9)	2.96	0.09					
Sexual abuse	5.0 (1.9)	4.4 (1.9)	2.04	0.16					
Emotional neglect	11.7 (5.2)	10.2 (4.2)	0.67	0.42					
Physical neglect	7.9 (3.4)	6.7 (2.3)	3.46	0.07					
Stressful life events (SLE) and Coping Strategies in Stressful Situations (CISS)									
Stressful life events before	3.2 (1.9)	2.2 (2.0)	5.24	0.02	3.6 (2.3)	2.3 (2.1)	3.40	0.07	0.29
Task‐oriented	52.1 (9.2)	50.7 (11.3)	0.46	0.50	55.6 (9.3)	49.0 (12.4)	4.99	0.03	0.55
Emotion‐oriented	45.2 (11.8)	43.6 (13.6)	1.51	0.22	42.6 (12.2)	38.7 (10.7)	1.29	0.26	0.17
Avoidance	40.2 (7.2)	39.2 (9.5)	0.26	0.61	40.6 (8.6)	38.0 (6.5)	1.49	0.23	0.37
Distraction	17.9 (5.4)	17.6 (5.3)	0.14	0.71	17.1 (5.7)	15.6 (4.1)	1.03	0.31	0.53
Social diversion	15.2 (4.1)	14.1 (5.4)	1.27	0.26	16.4 (4.2)	15.5 (5.4)	0.73	0.40	0.86

*Note:* Scores are indicated in mean (standard deviation). Bold values indicate significant values.

Abbreviations: AD = antidepressants; AE = antiepileptics; AP = antipsychotics; FAST = Functional Assessment Short Test; HDRS = Hamilton Depression Rating Scale; LI = lithium; YMRS = Young Mania Rating Scale.

**TABLE 4:** Cognition in affected monozygotic twins with and without illness relapse.

	Baseline				Follow‐up				Group‐by‐time interaction
	Affected relapsers (*n* = 67)	Affected non‐relapsers (*n* = 45)	*F*/*χ*	*p*	Affected relapsers (*n* = 52)	Affected non‐relapsers (*n* = 15)	*F*/*χ*	*p*	*p*
Emotional cognition									
DotProbe Faces Task									
Masked fear	−24.1 (64.8)	−32.9 (92.9)	0.31	0.58	−8.9 (67.3)	−22.0 (80.2)	0.40	0.53	0.89
Masked happy	11.5 (97.7)	27.7 (81.4)	0.73	0.40	40.2 (252.1)	−4.5 (74.0)	0.38	0.60	0.39
Unmasked fear	−13.3 (80.1)	−25.0 (90.7)	0.44	0.51	−41.8 (160.3)	−28.2 (55.0)	14	0.71	0.58
Unmasked happy	−15.6 (112.1)	23.5 (136.1)	2.39	0.13	28.4 (216.1)	−11.7 (88.1)	0.90	0.35	0.19
Facial Expression Recognition Test									
Discrimination accuracy (*d*')									
Positive emotions	0.5 (0.1)	0.5 (0.1)	0.87	0.35	0.5 (0.1)	0.5 (0.1)	0.11	0.92	0.28
Negative emotions	0.4 (0.1)	0.4 (0.1)	2.25	0.12	0.4 (0.1)	0.4 (0.1)	0.38	0.54	0.18
Response time, *z*									
Positive emotions	−0.2 (1.2)	−0.5 (1.5)	0.67	0.42	−0.1 (0.6)	−0.4 (0.7)	3.14	0.08	0.48
Negative emotions	−0.1 (1.0)	−0.4 (1.3)	1.88	0.17	−0.1 (0.8)	−0.6 (1.0)	3.57	0.08	0.63
Emotional Categorization and Recall Task									
Categorization accuracy (1 = 100%)									
Positive words	0.9 (0.1)	0.9 (0.1)	0.60	0.44	0.9 (0.1)	0.9 (0.1)	0.29	0.60	0.11
Negative words	0.9 (0.1)	0.9 (0.1)	0.05	0.82	0.9 (0.1)	1.0 (0.0)	2.59	0.11	0.32
Mean response time categorization, *z*									
Positive words	0.4 (1.1)	0.8 (1.2)	1.87	0.18	0.5 (1.3)	1.2 (1.7)	2.31	0.13	0.45
Negative words	0.4 (1.1)	0.6 (1.0)	0.37	0.54	0.4 (1.4)	0.6 (1.1)	0.09	0.77	0.97
Number of words recalled									
Positive words	3.4 (2.3)	3.1 (2.3)	0.55	0.46	3.7 (2.4)	2.7 (1.5)	2.52	0.12	0.33
Negative words	2.9 (2.2)	2.7 (1.9)	0.72	0.40	2.3 (2.8)	2.6 (1.6)	0.87	0.36	0.36
Social Scenarios Task									
Emotion reactivity									
Positive emotions	75.8 (16.5)	77.2 (13.5)	0.12	0.73					
Negative emotions	75.0 (16.0)	73.6 (18.0)	0.43	0.51					
Emotion downregulation									
Positive emotions	20.3 (18.1)	21.1 (19.7)	0.03	0.87					
Negative emotions	26.1 (18.6)	27.9 (19.3)	0.24	0.63					
Non‐emotional cognition									
TMT‐A, s	31.9 (13.0)	28.3 (8.6)	2.42	0.12	31.3 (12.0)	30.1 (7.7)	0.06	0.81	0.56
TMT‐B, s	80.1 (36.9)	80.1 (40.0)	0.00	0.99	76.8 (32.3)	67.0 (22.5)	0.12	0.73	0.27
SCIP total	−0.7 (1.3)	−1.0 (1.7)	1.57	0.21	−0.6 (1.6)	−1.3 (1.7)	2.62	0.11	0.52
VLT‐I^a^	−0.2 (1.2)	−0.6 (1.6)	1.28	0.26	−0.3 (1.4)	−0.8 (1.5)	1.55	0.22	0.36
WMT^a^	−0.5 (1.2)	−0.8 (1.6)	2.24	0.14	−0.4 (2.0)	−0.9 (1.8)	0.06	0.81	0.57
VFT^a^	−0.4 (1.3)	−0.5 (1.2)	0.27	0.61	−0.5 (1.1)	−1.0 (1.0)	2.08	0.16	0.47
VLT‐D^a^	−0.4 (1.1)	−0.5 (1.5)	0.06	0.80	−0.5 (1.4)	−0.7 (1.3)	0.21	0.65	0.15
PST^a^	−0.5 (1.5)	−0.5 (1.8)	0.08	0.78	−0.2 (1.0)	−0.5 (0.8)	1.15	0.29	0.80

*Note:* Scores are indicated in mean (standard deviation). Bold values indicate significant values.

^a^ Number of SD from expected scores based on sex, age, and years of education.

### 4.3 Demographics and Clinical Characteristics Over the Follow‐Up Time

Among unaffected twins with both baseline and follow‐up assessments, there were no significant group differences nor changes over time in mood symptoms, functioning, or coping (*p*s ≥ 0.07).

Regarding affected twins with cognitive assessments at both timepoints, relapsers were older (*p* = 0.02) and reported more stressful life events (*p* < 0.01). There was a significant group‐by‐time interaction effect for subsyndromal depressive symptoms (*F* (1, 84.31) = 4.67, *p* = 0.03), driven by symptom decrease in non‐relapsers only (*p* < 0.01). No group effects were observed for subsyndromal mania or anxiety or functioning (*ps* ≥ 0.06).

### 4.4 Aim 1. Is Baseline Cognition Associated With Subsequent Onset or Relapse Risk?

For unaffected twins, greater baseline vigilance toward supraliminal (consciously processed) happy faces predicted lower risk of illness onset (Exp(B) = 0.995, CI [0.990; 1.000], *p* = 0.03) (see Table 5). In contrast, subliminal and supraliminal vigilance toward fearful faces and subliminal vigilance toward happy faces were not significantly related to illness onset (*ps* ≥ 0.056). Performance on the facial expression recognition (*ps* ≥ 0.10), emotional categorization or recall of self‐referential words (*ps* ≥ 0.31) or emotional reactivity or downregulation during social scenarios (*ps* ≥ 0.24) was also unrelated to risk of illness onset. Better verbal fluency was unexpectedly linked to higher onset risk (Exp(B) = 1.589, CI [1.204; 2.097], *p* = 0.001) (see Table 5). No other non‐emotional cognitive markers predicted onset (*ps* ≥ 0.06).

**TABLE 5:** Baseline cognitive variables associated with future mood disorder events.

	Adjusted HR	Confidence intervals	*p*
Risk of onset in unaffected twins			
VFT	1.589	[1.204–2.097]	0.001
Unmasked happy faces	0.995	[0.990–1.000]	0.03
Risk of relapse in affected twins			
Unmasked happy faces	0.997	[0.995–0.999]	0.003
Risk of a mood episode event across the whole sample			
Unmasked happy faces	0.997	[0.995–0.999]	0.001

*Note:* This table reports only significant associations.

Abbreviation: HR = hazard ratio.

For affected twins, greater vigilance toward supraliminal happy faces at baseline predicted lower relapse risk (Exp(B) = 0.997, CI [0.995; 0.999], *p* < 0.01) (see Table 5). No other emotional or non‐emotional cognitive variables were associated with relapse (ps ≥ 0.09).

Across all groups, greater vigilance to supraliminal happy faces predicted reduced overall risk of a mood episode (Exp(B) = 0.996, CI [0.994; 0.998], *p* < 0.001) (see Table 5). No other emotional or cognitive variables showed significant associations (ps ≥ 0.10). For results regarding covariates, see Supporting Information.

### 4.5 Aim 2. Are Mood Episodes Associated With Change in Cognition Over Time?

**FIGURE 2:**
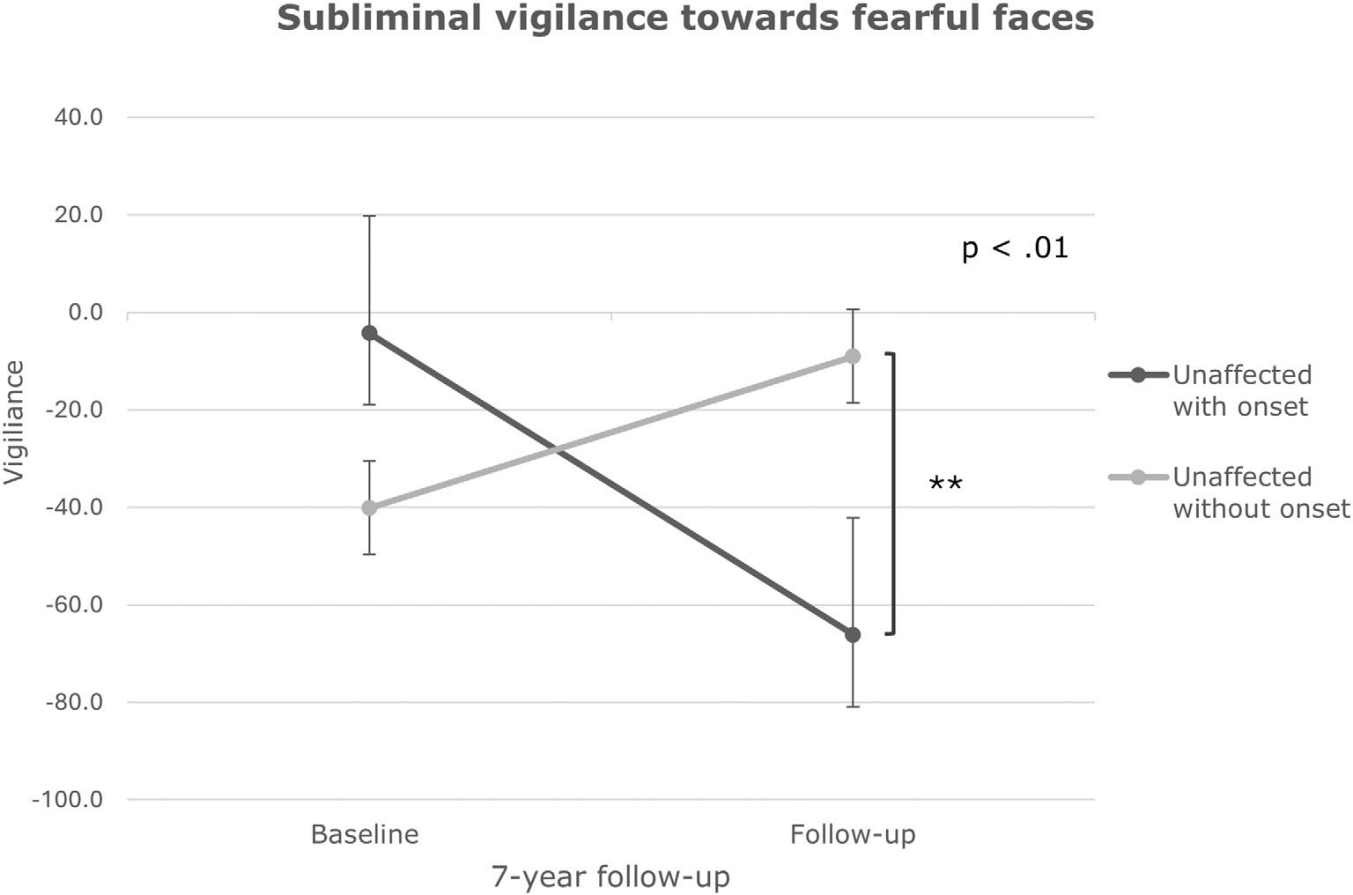
Subliminal vigilance toward fearful faces over time in unaffected monozygotic twins with and without onset. *p* values are presented for the significant main effect of the group across both time points; asterisks denote significant pairwise comparisons. (**p < 0.01). Error bars indicate the standard error of the mean.

**FIGURE 3:**
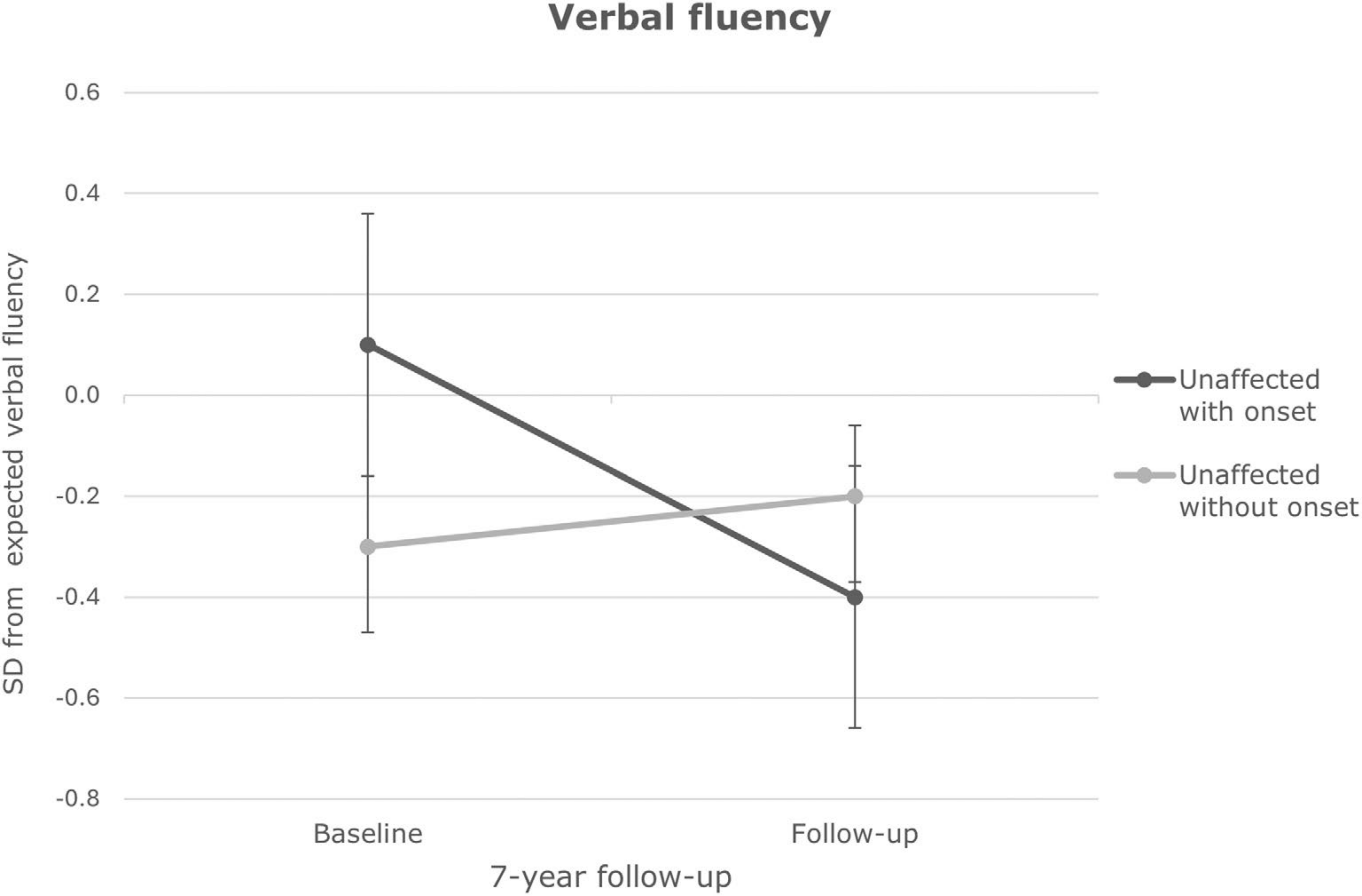
Verbal fluency changes over time in unaffected monozygotic twins with and without subsequent onset. Error bars indicate the standard error of the mean. No significant difference in verbal fluency was observed at baseline (*p* = 0.15) or follow‐up (*p =* 0.38); however, a significant group‐by‐time interaction was observed (*p =* 0.02). SD = standard deviation.

Among unaffected twins, those who subsequently developed illness onset showed increasing avoidance of (as reflected by 'more negative' vigilance scores to) subliminal fearful faces over time (group × time: *F* (1, 68.35) = 12.41, *p* < 0.001, partial *η* ^2^ = 0.15; estimated difference in change = 89.99, 95% CI [39.01; 140.96]; within‐group change: *p* = 0.03), whereas those who remained healthy showed reduced avoidance (*p* = 0.02) (see Figure 2). Verbal fluency declined over time in those with onset (group × time: *F*(1, 59.43) = 7.64, *p* = 0.008, partial *η* ^2^ = 0.11; estimated difference in change = 0.70, 95% CI [0.19;1.21]; within‐group change: *p* < 0.001), but remained stable in others (*p* = 0.51) (Figure 3). No other group or group‐by‐time differences were observed between those with and without illness onset (*ps* ≥ 0.07).

Among affected twins, no significant group or group‐by‐time effects were found for emotional (*ps* ≥ 0.08) or non‐emotional (*ps* ≥ 0.10) cognition (Table 4).

## 5 Discussion

This seven‐year prospective study examined the relationship between cognition and illness progression in monozygotic twins with mood disorders (MD), their high‐risk monozygotic twins, and low‐risk twins. We first assessed whether baseline cognition predicted illness onset or relapse. Greater attentional vigilance toward supraliminal happy faces was associated with reduced risk of both onset and relapse. In contrast, better verbal fluency was unexpectedly linked to increased risk of illness onset. Second, we examined whether mood episodes were associated with cognitive changes over time. Unaffected twins who developed a mood disorder over the follow‐up time showed a decline in verbal fluency, whereas those who remained unaffected showed stable performance. Additionally, illness onset was linked to increased avoidance of subliminal fearful faces over time, while avoidance decreased in those who remained healthy. No differential cognitive changes were observed among affected twins with and without relapse.

The protective effect of vigilance to consciously processed happy faces aligns with cognitive theories of depression, which emphasize the role of negative attentional biases in symptom onset and maintenance [53, 54, 55]. Indeed, studies have consistently demonstrated that individuals with mood disorders exhibit greater attention to—and difficulties disengaging attention from—negative stimuli as well as less attention to positive stimuli [56], which are associated with an increased risk of relapse [57, 58]. While positive biases have been associated with (hypo)manic risk in BD [24, 59], reducing negative biases through interventions that involve cognitive restructuring may help prevent relapse or onset [60, 61].

Unexpectedly, avoidance of subliminal fearful faces increased over time in those who developed illness. Although this contrasts prior dot‐probe findings in MDD [62], it may reflect maladaptive emotion regulation, such as experiential avoidance—a known risk factor for mood symptoms [63, 64]. Our previous work showed gaze aversion in early BD [65], consistent with this pattern. Indeed, avoidance is considered a maladaptive emotion regulation strategy associated with poor long‐term mental health and depressive symptoms [19]. The increased avoidance of subliminal fearful faces over time in unaffected twins with subsequent onset occurred in the absence of self‐reported avoidant coping strategies, suggesting a discrepancy between conscious and non‐conscious emotion processing. Thus, it is plausible that individuals at risk of mood disorders are more likely to engage in implicit avoidant coping strategies and disengage from negatively laden stimuli in an attempt to manage their emotions, which may paradoxically increase the frequency and intensity of depressive symptoms, contributing to illness onset. This supports early intervention to reduce negative biases and promote adaptive regulation strategies (e.g., cognitive reappraisal) in at‐risk individuals. However, this increased avoidance could also reflect more general cognitive disengagement or reduced affective salience rather than emotion regulation per se. As we did not include psychophysiological measures (e.g., galvanic skin response and pupil dilation) to corroborate implicit regulation processes, these interpretations remain tentative.

The finding that superior verbal fluency predicted illness onset was counterintuitive, as prior work linked poorer executive function with higher risk [66, 67]. However, some cohort studies have associated higher premorbid cognition with BD and MDD onset [68, 69, 70, 71]. Although speculative, this association may possibly be due to increased vulnerability to rumination [72] or alternatively a proxy for verbal overproduction or hyper‐association tendency, consistent with a subtype of at‐risk individuals who are high‐functioning but nonetheless vulnerable. Notably, verbal fluency declined post‐onset, which contrasts with prior demonstration of stable subtle impairment in set‐shifting and verbal learning and memory following onset in a 2‐year follow‐up study [73]. The decline in verbal fluency may reflect adverse effects of illness onset, potentially indicating a scar effect. Indeed, verbal fluency impairments have been associated with cortical thinning in prefrontal and temporal regions in mood disorders [74, 75] and in neurodegenerative conditions [76]. Although treatment effects cannot be fully excluded, few newly affected individuals received medication (Table S1), making this unlikely.

Strengths of the study include its prospective twin design, use of Danish registries, long follow‐up, and assessment of both emotional and non‐emotional cognition. The sample of discordant twins was modest (*n* = 63), limiting power for subgroup analyses. Thus, the lack of significant trajectory differences between groups in other cognitive domains, such as executive function and verbal memory, should be interpreted with caution, as these null results may reflect insufficient statistical power rather than true absence of effects. Future studies with larger samples are needed to clarify whether more subtle cognitive changes are present in these domains among individuals at familial risk and those affected with mood disorders. However, the unique design enabled examination of cognitive trajectories before and after illness onset. Concerning the cognitive retest analyses, it is a limitation that 39% did not participate in the full follow‐up assessment. Nevertheless, linear mixed models are well‐suited for unbalanced longitudinal design, as they can accommodate varying numbers of observations per participant. Further, comparative analyses indicated that participants missing at follow‐up did not differ significantly from those with complete data sets (see Supplement). Moreover, BD and MDD cases were combined to maximize sample size, which may have obscured disorder‐specific cognitive differences (e.g., negative bias prevalent in MDD). In addition, information on the number of types of episodes was not systematically collected at follow‐up, limiting further analyses stratifying BD versus MDD or examining diagnostic conversions. Future studies with larger, stratified samples should address these distinctions. Finally, the SCIP, while psychometrically strong [77], may not detect subtle cognitive changes. Future studies should use more comprehensive cognitive test batteries to better capture domain‐specific changes.

In conclusion, our findings indicate that positive attentional biases may protective against illness onset and relapse, while increasing avoidance of fearful stimuli signal heightened risk. Additionally, greater baseline verbal fluency may predict onset, with subsequent decline potentially reflecting a scar effect of illness. These findings underscore the importance of early identification of cognitive‐emotional vulnerabilities and support future trials targeting emotion regulation and cognition in high‐risk individuals.

## Author Contributions

**K.W.M.:** conceptualization, methodology, investigation, writing – original draft, supervision, project administration, writing – review and editing. **H.L.K.:** formal analysis, investigation, data curation, writing – original draft, writing – review and editing, visualization, supervision. **S.L.:** formal analysis, data curation, writing – original draft, writing – review and editing, visualization. **A.S.:** formal analysis, writing – review and editing. **L.K.:** conceptualization, methodology, writing – review and editing. **M.V.:** conceptualization, methodology, investigation, writing – review and editing, supervision, project administration, funding acquisition.

## Ethics Statement

The authors assert that all procedures contributing to this work comply with the ethical standards of the Danish National Board of Health, the Data Protection Agency (Approval No. 2014–331‐0751), and the Regional Ethics Committee (Approval Nos. H‐3‐2014‐003, H‐22022286) and Institutional committees on human experimentation and with the Helsinki Declaration of 1975, as revised in 2008.

## Conflicts of Interest

K.W.M. has received honoraria from Angelini, Lundbeck and Gedeon Richter in the past three years. H.L.K. has received honoraria from Lundbeck. L.K. has within recent three years received honoraria from Lundbeck and Teva. The remaining authors have nothing to declare. M.V. has received honoraria from Lundbeck, Johnson & Johnson and Eli Lilly. The remaining authors report no conflicts of interest.

## Supporting information


**Data S1:** Supporting Information.

## Data Availability

The data that support the findings of this study are available on request from the corresponding author. The data are not publicly available due to privacy or ethical restrictions.
