# Supplementary material for: Associations Between Cognitive Functions and Subsequent Mood Disorder Prognosis in Low‐Risk, High‐Risk and Affected Monozygotic Twins: A Seven‐Year Follow‐Up Study
Source: Acta Psychiatr Scand. 2025 Aug 20;153(5):432–48. doi: 10.1111/acps.70025 (PMC13050597; doi:10.1111/acps.70025)
Supplement: Supplementary file 1 — Data S1: Supporting Information. [file ACPS-153-432-s001.docx]

**Supplementary material**

**Methods**

**Assessment of emotional cognition**

Participants completed a comprehensive emotional cognitive test battery. Emotional processing was assessed using three computerized Oxford Emotional Test Battery tasks: Facial Expression Recognition, Emotional Categorization and Recall, and The Faces Dot-Probe (P1Vital® Oxford Emotional Test Battery (ETB), 2017). Emotion reactivity and regulation were investigated at baseline using the Social Scenarios Task (Kjærstad et al., 2016).

***Facial Expression Recognition Test***

During the Facial Expression Recognition Task, 250 faces from the Pictures of Facial Affect Series (Ekman & Friesen, 1976) showing six basic emotions — anger, disgust, fear, happiness, sadness, or surprise — were presented randomly. The faces were modulated at 10% intensity levels, ranging from neutral (0%) to a fully emotional face (100%), and displayed for 500 ms, followed by a black screen. Participants were asked to identify the facial emotional expression of each face as fast and correctly as possible by pressing the matching key labeled on the computer keyboard.

***Emotional Categorization and Recall Task***

In the Emotional Categorization and Recall Task, participants were shown 45 positive and 45 negative personality characteristics retrieved from Anderson’s list of personality trait words (Anderson, 1968) translated into Danish. Participants were required to classify the traits as either favorable or unfavorable as rapidly and correctly as possible in a self-referential manner. Self-reference was established prompting participants to imagine overhearing others describe them using these trait words. In a recall task conducted 15 minutes after task completion, participants were asked to list all the words they could remember from the categorization task within five minutes.

***The Faces Dot-Probe Task***

During the Faces Dot-Probe Task, participants were shown pairs of happy-neutral, fearful-neutral, and neutral-neutral faces horizontally, either unmasked for 100 ms (supraliminal processing) or masked for 17 ms (subliminal processing). One of the faces was swiftly interchanged with two dots displayed either vertically (:) or horizontally (··). Participants were instructed to evaluate the orientation of the dots as rapidly and correctly as possible by pressing matching labeled keys. A total of 32 trials across six conditions were administered during the task: masked or unmasked happy-neutral, fearful-neutral, or neutral-neutral. Eight blocks of unmasked faces and eight blocks of masked faces, each consisting of 12 trials, including all face-pair types, were administered in an alternating order.

***Social Scenarios Task***

In the Social Scenarios task, participants were asked to read accounts of nine social scenarios displayed on a computer screen. Each scenario consisted of eleven sentences describing the social scenario and ten related self-belief statements. The first scenario was neutral, while the remaining eight were either positive or negative. Before the scenarios, participants were instructed to respond intuitively or try to down-regulate their emotional reactions. Accordingly, they were presented with five conditions: “react neutral,” “react positive,” “react negative," "down-regulate positive,” and "down-regulate negative.” No specific strategies were provided to ensure that participants used their habitual emotion regulation techniques. After reviewing the self-belief statements, participants were asked to evaluate their level of discomfort or pleasure using a 100-point VAS scale, resulting in a total of 10 emotion ratings. As some scenarios contain romantic elements, participants’ sexual orientation was determined before task commencement, and the appropriate version of the task was administered.

**Results**

**Comparative analyses of drop-outs and follow-up participants**

Comparative analyses of participants with only baseline assessment and those with baseline and follow-up assessments revealed no significant differences in age, predicted full-scale IQ, years of education, subsyndromal depressive and manic symptoms, overall functioning, or number of mood episodes (*ps* ≥ .18), except for verbal full-scale IQ, for which low-risk twins participating solely at baseline had significantly lower IQ than those who participated at both time points (*p* = .02).

**Demographics and clinical characteristics**

***Affected, high-risk and low-risk twins at baseline***

Affected, high-risk, and low-risk twins were comparable in sex (*p* = .40), age (*p* = .87), years of education (*p* = .12), and verbal full-scale IQ (*p* = .26) (see Table S1). Affected twins had more residual depressive symptoms than both high-risk and low-risk twins (*ps* < .001), with no difference between high- and low-risk groups (*p =* .18). No group differences were found in subsyndromal manic symptoms (*p* = .22).

The FAST measures showed significant group differences (*p* *≤* .04). Affected twins presented with poorer overall functioning, as well as within the subdomains of autonomy, occupation, cognition, and relationships, compared to both high-risk and low-risk twins (*ps ≤* .01), with no difference between high- and low-risk groups (*ps* ≥ .66). Affected twins had significantly poorer financial functioning compared to high-risk twins (*p* = .02), while low-risk twins were comparable to both groups (*ps* ≥ .10). Affected twins had significantly more leisure impairment than low-risk twins (*p* = .01), though high-risk twins did not differ from either group (*ps* ≥ .06).

Affected twins reported significantly higher levels of anxiety (STAI trait/state, *ps ≤* .01) and neuroticism (*ps* < .001) compared both high-risk and low-risk twins, with no difference between high- and low-risk twins (*ps* ≥ .26). No group differences in reported stressful life events during lifetime were observed (*p* = .07). However, affected twins reported greater use of emotion-oriented coping and distraction in stressful situations compared to both low-risk and high-risk twins (*ps* *≤* .01), who conversely did not differ in the use of emotion-oriented coping (*ps* ≥ .71). A greater use of social diversion was reported in affected twins than low-risk twins (*p* = .003). In contrast, low-risk, high-risk, and affected and high-risk reported the same level of use of social diversion (*ps* ≥ .07). High-risk twins reported more use of task-oriented coping compared to affected twins (*p* = .004), while low-risk twins did not differ from either group (*ps* ≥ .09). No group differences were observed for the reported use of avoidant coping (*p* = .18).

The groups differed significantly in total childhood adversity, emotional abuse, sexual abuse, emotional neglect, and physical neglect (*ps ≤* .05)*.* Affected twins and their high-risk twins had experienced similar levels of emotional abuse, emotional neglect, and physical neglect (*ps* ≥ .60), whereas low-risk twins reported significantly fewer of these adversities (ps *≤* .05). Affected twins had experienced significantly more sexual abuse than low-risk twins (p = .003), but no difference was observed between affected vs. high-risk or high-risk vs. low-risk (*ps* ≥ .15). No group differences were observed for physical abuse (p = .48).

***Changes over time for affected, high-risk and low-risk twins***

Analyses of change in subsyndromal symptoms revealed a main effect of group (HDRS: *F* (2, 142.09) = 14.56, p < .001; YMRS: *F* (2, 146.29) = 3.08, *p* = .049), but no group-by-time interactions (see Table S1). Affected twins generally presented with more subsyndromal mood symptoms than low-risk twins across both timepoints (*ps* ≤ .02). Affected twins also had more subsyndromal depressive symptoms than high-risk twins (*p* < .001), but similar subsyndromal manic symptom levels (*p* = .50). No significant difference in subsyndromal manic symptoms was observed between high- and low-risk twins (*p* = .08).

For overall functioning (FAST), a main effect of group was observed (*F* (2, 158.80) = 26.21, *p* < .001), with affected twins generally showing greater impairment than both high-risk and low-risk twins across both timepoints (*ps* < .001), while high- and low-risk twins were comparable (*p* = .41). FAST subdomains showed main effects of group but no group-by-time interactions for autonomy (*p* = .01), cognition (*p* < .001), relationships (*p* < .001), occupation (*p* < .001), and financial functioning (*p* = .04). Affected twins had greater impairment in cognition, relationships, and occupation compared to both high- and low-risk twins (*ps* ≤ .004), while high- and low-risk twins were comparable (*ps* ≥ .20). Affected twins also had greater impairment in autonomy and financial functioning than high-risk twins (*ps* ≤ .01), while affected and low-risk twins and high- and low-risk twins were comparable (*ps* ≥ .21). No significant effects were found for FAST leisure (*ps* ≥ .07).

For trait and state-related anxiety, a main effect of group was found (STAI trait: *F* (2, 137.96) = 27.29, *p* < .001; STAI state: *F* (2, 159.52) = 11.88, *p* < .001), but not group-by-time interaction effects. Affected twins generally showed higher both trait- and state-related anxiety than high- and low-risk twins across both timepoints (*ps* ≤ .02), while high- and low-risk twins were comparable (*ps* ≥ .45).

A main effect of group, but not group-by-time interaction, was observed for lifetime stressful events (*F* (2, 159.59) = 4.46, *p* = .01), with affected twins reporting generally more events than low-risk twins across both timepoints (*p* = .01), while affected and high-risk twins (*p* = .07), and high-risk and low-risk twins (*p* = .21) reported a similar number of events.

For coping strategies, a main effect of group, but no group-by-time interactions, was found for task-oriented coping (*F* (2, 166.92) = 3.81, *p* = .02), emotion-oriented coping (F (2, 124.97) = 11.81, *p* < .001), and distraction (F (2, 152.50) = 5.43, *p* = .01). Affected twins used more emotion-oriented coping (*ps* ≤ .01) and distraction (*ps* ≤ .02) than both high- and low-risk twins, who instead reported similar usage of the strategies (ps ≥ .89). High-risk twins used more task-oriented coping than affected twins (*p* = .01), but no differences were found between affected and low-risk twins (*p* = .25) or high- and low-risk twins (*p* = .34). No group or group-by-time interactions were observed for avoidance (*p* = .52) or social diversion (*p* = .15).

**Associative risk of covariates**

**Overall risk of an event across groups as a function of non-emotional cognitive measures**

However, age was related to a higher risk of a mood disorder event during follow-up, showing hazard ratios (HR) between 1.032 and 1.038, *p*-values ranging from *p* = .03 to *p* = .76, with seven out of eight models being significant (*ps* ≤ .04). Interestingly, for models including TMT-A (Exp(b) = 1.031, CI [1.002-1.062], *p =* .002) and TMT-B (Exp(b) = 1.113, CI [1.047-1.1184], *p* < 0.001) subsyndromal depressive symptoms were significantly associated with risk of a mood disorder event. This was, however, not found in the six models featuring the SCIP subtests or total SCIP score (*p* ≥ .16).

**Overall risk of an event across groups as a function of emotional cognitive measures**

Higher subsyndromal depressive symptoms at baseline were related to an increased risk of a mood disorder event, likely reflecting a greater risk of a mood disorder event in the affected group, showing hazard ratios between 1.104 and 1.150, *p*-values ranging between *p* < .001 to *p* = .73, with 16 out of 18 models being significant (*ps* ≤ .01). Higher age was related to an increased risk of a mood disorder event, revealing hazard ratios between 1.034 and 1.036, with *p*-values ranging between *p =* .03 to *p* = .15, with four out of 18 significant models (*ps* ≤ .05). Sex was not a significant covariate in any model (*ps* ≥ .59)

**Risk of relapse in affected twins as a function of non-emotional cognition**

Higher age was associated with a higher risk of relapse during follow-up, showing hazard ratios (HR) between 1.039 and 1.043 and *p*-values ranging from *p* = .01 to *p* = .72, with five out of eight models being significant (*p* ≤ .03). Similarly, the number of previous mood episodes was significantly associated with a greater risk of relapse during follow-up, showing hazard ratios (HR) between 1.023 and 1.029 and *p*-values ranging from *p =* .01 to *p* = .07, with seven out of eight models being significant (*p* ≤ .04). No other covariates were significantly associated with relapse during follow-up.

**Risk of relapse in affected twins as a function of emotional cognition**

Age was associated with a higher risk of relapse during follow-up, showing hazard ratios (HR) between 1.004 and 1.045 and *p*-values ranging from *p* = .02 to *p* = .77, with 10 out of 18 models being significant (*p* ≤ .049). In the model including supraliminal attention toward happy faces, male sex was significantly associated with a lower relapse risk (Exp(b) = .387, CI [.174; .862], *p* = .02). No other covariates were significantly associated with relapse during follow-up.

**Risk of onset in unaffected twins as a function of non-emotional cognition**

No covariates were significantly associated with onset during follow-up.

**Risk of onset in unaffected twins as a function of emotional cognition**

No covariates were significantly associated with onset during follow-up.

**Table S1: Demographics and clinical variables for affected, high-risk, and low-risk twins**

|  | Baseline | | | | | | Follow-up | | | | | | Group-by-time interaction |
| --- | --- | --- | --- | --- | --- | --- | --- | --- | --- | --- | --- | --- | --- |
|  | Affected  (AF)  (*n* = 115) | High-risk (HR)  (*n* = 49) | Low-risk  (LR)  (*n* = 40) | F/ χ^2^ | P-value | Pairwise comparisons | Affected  (AF)  (*n* = 71) | High-risk (HR)  (n = 34) | Low-risk (LR)  (n = 25) | F/ χ^2^ | P-value | Pairwise  comparisons |  |
| Demographics | | | | | | | | | | | | |  |
| Sex, n (% female) | 82 (71) | 33 (67) | 32 (80) | 1.19 | .40 |  |  |  |  |  |  |  |  |
| Age | 36.1  (8.8) | 36.9  (9.6) | 37.1  (9.2) | 0.14 | .87 |  |  |  |  |  |  |  |  |
| Premorbid IQ | 113.5 (6.4) | 112.4 (6.7) | 114.0 (5.7) | 1.36 | .26 |  |  |  |  |  |  |  |  |
| Education years | 14.5 (3.3) | 15.7 (3.1) | 15.3 (2.6) | 2.14 | .12 |  |  |  |  |  |  |  |  |
| Functioning | | | | | | | | | | | | |  |
| FAST total | 13.4 (11.5) | 3.3 (3.7) | 3.4 (5.7) | 30.30 | **< .001** | **AF >**  **HR & LR** | 22.9 (13.7) | 10.4 (7.2) | 15. 2 (10.3) | 15.85 | **< .001** | **AF > HR & LR** | .24 |
| FAST autonomy | 0.9 (1.6) | 0.4 (1.0) | 0.2 (0.5) | 5.11 | **.01** | **AF >**  **HR & LR** | 2.4 (2.5) | 1.2 (1.7) | 2.0 (1.9) | 3.83 | **.03** | **AF > HR**  **AF = LR**  **HR = LR** | .27 |
| FAST occupation | 5.5 (6.5) | 0.2 (0.6) | 0.5 (2.4) | 26.23 | **< .001** | **AF >**  **HR & LR** | 7.6 (6.8) | 1.6 (3.6) | 2.8 (4.5) | 15.88 | **< .001** | **AF > HR & LR** | .73 |
| FAST cognition | 3.0 (2.6) | 1.2 (1.6) | 1.3 (1.9) | 16.16 | **< .001** | **AF >**  **HR & LR** | 4.7 (3.3) | 2.2 (1.8) | 3.4 (3.1) | 11.63 | **< .001** | **AF > HR**  **AF = LR**  **HR = LR** | .23 |
| FAST financial | 0.4 (1.0) | 0.2 (0.5) | 0.2 (0.6) | 3.26 | **.04** | **AF > HR**  **AF = LR HR = LR** | 0.7 (1.3) | 0.4 (0.7) | 0.8 (1.1) | 1.79 | .17 |  | .33 |
| FAST relationships | 2.7 (3.1) | 1.0 (1.5) | 1.0 (1.8) | 11.48 | **< .001** | **AF >**  **HR & LR** | 5.4 (3.5) | 3.4 (2.4) | 4.0 (2.9) | 5.80 | **.004** | **AF > HR,**  **AF = LR**  **HR = LR** | .89 |
| FAST leisure | 0.9 (1.4) | 0.5 (0.8) | 0.3 (0.9) | 4.04 | **.02** | **AF > LR,**  **AF = HR**  **HR = LR** | 2.0 (1.7) | 1.7 (1.3) | 2.3 (1.6) | 1.24 | .30 |  | .18 |
| Clinical variables | | | | | | | | | | | | |  |
| UD, n (%) | 84 (73) | 38 (78) |  |  |  |  |  |  |  |  |  |  |  |
| BD, n (%) | 31 (27) | 11 (22) |  |  |  |  |  |  |  |  |  |  |  |
| BD type II, n (% of BD) | 9 (29) |  |  |  |  |  |  |  |  |  |  |  |  |
| Age at onset | 23.4 (7.7) |  |  |  |  |  |  |  |  |  |  |  |  |
| Total episodes | 4.9 (9.5) | 0.0 (0.0) | 0.0 (0.0) |  |  |  |  |  |  |  |  |  |  |
| Hospitalizations | 2.3 (10.3) | 0.0 (0.1) | 0.0 (0.0) |  |  |  |  |  |  |  |  |  |  |
| Suicide attempts | 0.9 (4.9) | 0.0 (0.1) | 0.0 (0.0) |  |  |  |  |  |  |  |  |  |  |
| HDRS | 4.8 (3.6) | 2.7 (2.5) | 1.9 (2.1) | 16.25 | **< .001** | **AF >**  **HR & LR** | 4.4 (3.6) | 2.4 (2.8) | 2.5 (3.2) | 5.53 | **.01** | **AF >**  **HR & LR** | .54 |
| YMRS | 1.8 (2.1) | 1.5 (1.3) | 1.3 (1.5) | 1.54 | .22 |  | 1.1 (1.9) | 1.0 (2.1) | 0.1 (0.3) | 2.43 | .09 |  | .47 |
| STAI-state | 31.8 (7.5) | 28.8 (6.8) | 27.0 (6.9) | 7.58 | **< .001** | **AF >**  **HR & LR** | 35.1 (9.2) | 29.5 (6.7) | 28.9 (8.9) | 7.51 | **<.001** | **AF >**  **HR & LR** | .37 |
| STAI-trait | 41.5 (8.7) | 34.0 (5.4) | 34.2 (5.9) | 26.45 | **< .001** | **AF >**  **HR & LR** | 41.5 (8.5) | 34.5 (7.0) | 35.8 (7.0) | 11.80 | **<.001** | **AF >**  **HR & LR** | .78 |
| Neuroticism | 11.8 (5.5) | 7.4 (5.1) | 6.4 (4.2) | 20.60 | **< .001** | **AF >**  **HR & LR** |  |  |  |  |  |  |  |
| Medication, n (%) | | | | | | | | | | | | | |
| AD | 45 (39) | 1 (2) | 0 (0) |  |  |  | 23 (21) | 4 (8) | 0 (0) |  |  |  |  |
| AP | 18 (16) | 0 (0) | 0 (0) |  |  |  | 7 (6) | 0 (0) | 0 (0) |  |  |  |  |
| AE | 17 (15) | 0 (0) | 0 (0) |  |  |  | 8 (7) | 0 (0) | 0 (0) |  |  |  |  |
| LI | 12 (10) | 0 (0) | 0 (0) |  |  |  | 4 (4) | 0 (0) | 0 (0) |  |  |  |  |
| Childhood adversity (CTQ) | | | | | | | | | | | | |  |
| CTQ Total | 38.0 (12.0) | 36.8 (12.8) | 28.7 (6.7) | 5.62 | **.01** | **AF = HR**  **> LR** |  |  |  |  |  |  |  |
| Physical abuse | 5.8 (1.8) | 5.9 (2.1) | 5.3 (1.1) | 0.75 | .48 |  |  |  |  |  |  |  |  |
| Emotional abuse | 9.3 (4.4) | 8.9 (4.7) | 6.6 (2.5) | 3.78 | **.03** | **AF = HR**  **> LR** |  |  |  |  |  |  |  |
| Sexual abuse | 4.7 (1.9) | 4.3 (1.3) | 3.6 (1.0) | 5.02 | **.01** | **AF > LR**  **HR = LR**  **AF = HR** |  |  |  |  |  |  |  |
| Emotional neglect | 11.2 (5.1) | 10.7 (5.5) | 7.6 (2.5) | 5.31 | **.01** | **AF = HR**  **> LR** |  |  |  |  |  |  |  |
| Physical neglect | 7.4 (3.1) | 7.0 (2.6) | 5.8 (2.4) | 3.08 | **.050** | **AF = HR**  **> LR** |  |  |  |  |  |  |  |
| Stressful life events (SLE) and Coping Strategies in Stressful Situations (CISS) | | | | | | | | | | | | |  |
| SLE lifetime before | 2.8 (2.0) | 2.4 (1.9) | 1.9 (1.4) | 2.69 | .07 |  | 3.2 (2.3) | 2.3 (1.7) | 2.0 (1.5) | 4.51 | **.01** | **AF >**  **HR & LR** | .50 |
| Task-oriented | 51.8 (10.0) | 57.5 (11.0) | 55.2  (11.5) | 4.73 | **.01** | **HR > AF**  **LR = AF**  **HR = LR** | 53.6 (10.3) | 58.6 (9.3) | 55.8 (11.5) | 2.61 | .08 |  | .48 |
| Emotion-oriented | 44.8 (12.5) | 36.1 (12.2) | 36.7 (11.9) | 12.39 | **< .001** | **AF >**  **HR & LR** | 42.1 (12.1) | 34.4 (11.1) | 35.9 (10.7) | 6.23 | **.003** | **AF >**  **HR & LR** | .70 |
| Avoidance | 39.6 (8.2) | 37.5 (7.5) | 40.6 (8.9) | 1.73 | .18 |  | 39.7 (8.4) | 38.8 (8.6) | 36.4 (8.2) | 1.43 | .24 |  | .19 |
| Distraction | 17.7 (5.3) | 14.4 (4.9) | 15.0 (4.4) | 8.11 | **< .001** | **AF >**  **HR & LR** | 16.9 (5.4) | 15.1 (5.0) | 14.8 (4.3) | 2.02 | .14 |  | .31 |
| Social diversion | 14.7 (4.7) | 15.7 (4.3) | 17.5 (4.4) | 4.82 | **.01** | **AF > LR, HR = AF**  **LR = HR** | 15.8 (4.7) | 16.9 (4.3) | 15.5 (5.4) | .68 | .51 |  | .07 |

Values are indicated in mean (standard deviation) unless otherwise is specified. Bold values indicate significant values. Abbreviations: HDRS = Hamilton Depression Rating Scale; YMRS = Young Mania Rating Scale; FAST = Functioning Assessment Short Test; AD = antidepressants; AP = antipsychotics; AE = antiepileptics; LI = lithiu
